# The moderation effect of cognitive reserve on the longitudinal association between depression and cognitive performance among older adults

**DOI:** 10.1002/alz70860_097597

**Published:** 2025-12-23

**Authors:** Rabia Khalaila

**Affiliations:** ^1^ Zefat Academic College, Zefat, CA, Israel; University of California, San Francisco / Global Brain Health Institute, San Francisco, CA, USA

## Abstract

**Background:**

Cognitive decline and depression significantly affect the aging population, posing challenges for geriatric healthcare. Cognitive reserve (CR) is hypothesized to moderate these impacts by preserving cognitive function despite neuropathological changes.

**Objective:**

To examine the association between depression and cognitive decline and the moderating effects of cognitive reserve proxies (education, occupational complexity, and cognitive activities) over four years.

**Method:**

Utilizing data from 32,325 participants aged 50+ from the Survey of Health, Aging, and Retirement in Europe (SHARE), cognitive performance was assessed using memory, numeracy, and verbal fluency tests in baseline and follow up of 4 years. Depressive symptoms were measured with the EURO‐D scale, and CR was evaluated through educational attainment, occupational complexity, and engagement in cognitive activities. The moderation effects of CR on the relationship between depressive symptoms and cognitive performance were tested using bootstrapping with resampling strategies.

**Result:**

Lower and average levels of CR were associated with a stronger negative correlation between depressive symptoms and cognitive performance, while higher levels of CR showed no adverse effects. Education and engagement in cognitive activities significantly reduced the negative impacts of depression on cognitive functions, unlike occupational complexity, which showed no significant effect.

**Conclusion:**

Cognitive reserve plays a critical role in moderating the longitudinal effect of depression on cognitive performance among older adults. Enhancing CR through education and cognitive activities could mitigate the adverse effects of depression on cognitive health, highlighting the need for public health policies to incorporate CR‐enhancing strategies in later life.

